# Mineralized carbonates contribute to the millennial durability of Roman concrete

**DOI:** 10.1126/sciadv.aeb0754

**Published:** 2026-07-08

**Authors:** Xiaohong Zhu, Sejung Rosie Chae, Stuart McElhany, Chengyao Liang, Qi Zheng, Jiaqi Li, Veronica Fondi, Sergio Del Ferro, Ascanio Modena Altieri, Harrison P. Lisabeth, Arun Bhattacharjee, Bruce W. Fouke, Hans-Rudolf Wenk, Paulo J. M. Monteiro

**Affiliations:** ^1^ Department of Civil and Environmental Engineering, University of California, Berkeley, CA 94720, USA.; ^2^ State Key Laboratory of Bridge Safety and Resilience, Beijing University of Technology, Beijing, 100124, China.; ^3^ Department of Chemical Engineering, University of California, Berkeley, CA 94720, USA.; ^4^ Department of Civil and Environmental Engineering, University of Michigan, Ann Arbor, MI 48109, USA.; ^5^ Istituto Autonomo Villa Adriana e Villa d’Este, Ministero della Cultura, Tivoli, Roma, Italy.; ^6^ Lawrence Berkeley, National Lab, University of California, Berkeley, CA 94720, USA.; ^7^ Earth Science & Environmental Change, University of Illinois Urbana-Champaign, Urbana, IL 61801, USA.; ^8^ Department of Earth and Planetary Science, University of California, Berkeley, CA 94720, USA.

## Abstract

Roman concrete structures have remained serviceable for nearly two millennia and are widely regarded as outstanding examples of durable ancient engineering. Existing literature attributes Roman concrete longevity to the pozzolanic reaction that occurs between reactive volcanic ashes and lime. While the pozzolanic reaction is of fundamental importance, we argue that carbonation over a long period of time also substantially enhances the durability and potential self-healing properties of concrete. To validate this claim, a comprehensive analysis of the concrete used in a latrine included in the Canopus western substructures, in Hadrian’s Villa (Roman imperial residence, Tivoli, Italy, 2nd century AD), is performed using multiscale spectroscopic and tomographic approaches to uncover the mechanisms behind the resilience of these enduring structures. Microstructural evidence reveals that volcanic lava composed of leucite, analcime, and ferrian diopside was combined by the Romans with lime at a water-to-binder ratio of ~0.4 to 0.45 to mix the concrete. Calcite cementation in pore spaces and fractures served as the primary binding mineral phase, while the formation of a relatively minor amount of calcium-aluminum-silicate-hydrate precipitated around and between lava fragments to further enhance the integrity of concrete. Conversely, unhydrated calcium oxide reacted with atmospheric carbon dioxide and moisture to form volumetrically dominant calcite cements, creating the primary driver to reinforce structural strength and occlude porosity. This radiaxial fibrous calcite has the potential to mitigate environmental and mechanical stresses in modern concrete infrastructure and advance the development of sustainable and resilient construction materials in the future.

## Teaser

Mineralized carbonation created calcium carbonate networks that strengthened ancient Roman concrete over millennia.

## INTRODUCTION

Roman concrete not only bears witness to the rise and fall of the Roman empire but also preserves an indelible record of one of the most notable engineering advancements achieved throughout antiquity and the modern world. Unlocking now lost secrets developed by the Romans to enhance the durability of ancient concrete would revolutionize society’s ability to attain sustainable modern infrastructure development (*1*–*3*). Roman builders used different mix proportions to produce concrete strategically tailored for its specific use in marine (*4*–*6*) versus terrestrial (*7*–*9*) environments, optimizing their concrete-producing materials to suit specific functional requirements.

Iconic inland buildings, such as Trajan’s market, have been systematically studied (*7*, *10*), revealing that the durability of the concrete used in its construction stems from the formation of calcium-aluminum-silicate-hydrate (C-A-S-H) amorphous and crystalline phases and the autochthonous crystallization of durable minerals like strätlingite and hydrogarnet. These deposits toughen interfacial zones, inhibit crack propagation, and enhance fracture resistance (*7*, *10*). The pozzolanic reactivity of volcanic ash, sourced from the Pouzzolane Rosse pyroclastic flow, plays a pivotal role by dissolving silicate and alumina components that react with lime to form resilient binding phases (*7*). Similarly, a study of the Privernum city wall highlights the role of lime clasts in the self-healing attributes of Roman concrete (*9*). The recent study by Seymour *et al.* (*9*) claims that Roman builders used a “hot mixing” technique that generated high localized temperatures during lime hydration, which facilitated the formation of a gradient in hydration and carbonation around the lime clasts despite the potential challenges of elevated temperatures that may not have been ideal for construction. By contrast, marine Roman concrete used seawater to mix with volcanic ash, forming C-A-S-H and other mineral phases as the primary binding agents, resulting in a more complex matrix than those used for terrestrial Roman concrete (*4*–*6*, *11*).

Recently, there is a renewed interest in studying Roman concrete to guide the development of low-carbon cements (*6*). Many of these achievements are based on extensive previous studies on the long-term pozzolanic reaction between the volcanic ashes and the lime in Roman concrete. It is worth noting that Roman concrete can also provide insightful information on the long-term reaction with carbon dioxide (CO_2_; carbonation) of the mineral phases existing in the concrete matrix. This knowledge can provide insight into the long-term mineralogical evolution and natural carbonation of lime-based binders, which may inform conceptual strategies for durable low-clinker systems (*12*–*14*). The carbonation documented in Roman concrete would have advanced only gradually over centuries to millennia under diffusion-limited conditions, and such slow natural processes cannot be assumed to deliver substantial climate benefits within the operational life span of modern infrastructure. Thus, other than C-A-S-H formed, mineralogical analysis of some inland Roman concrete structures has suggested that calcite is the volumetrically dominant binding phase (*7*, *9*). Therefore, it becomes crucial to understand how calcium carbonate crystallization dynamics bind concrete together and contribute to its extraordinary durability. To address this, we studied the concrete from a latrine in the Canopus western substructures, a representative inland Roman concrete structure from the Hadrian’s Villa complex in Tivoli, Italy (2nd century AD). We used micro–computed tomography (μCT) with a pixel resolution down to 600 nm and nano-CT with a resolution of ~43 nm, in conjunction with backscattered scanning electron microscopy (BSEM) and multiscale spectroscopic analyses, to investigate the Canopus western substructure latrine concrete. These advanced imaging techniques allowed for an unprecedented, in-depth exploration of the calcite cementing mechanisms that enhance Roman concrete’s durability. By studying the hydration of lime clasts and their role in binding and formation processes of calcium carbonate, this study provides a comprehensive understanding of calcite’s durable mechanisms in Roman concrete. These insights offer valuable inspiration for developing innovative, sustainable concrete-based construction materials that incorporate self-healing properties to enhance modern infrastructure resilience and longevity.

## RESULTS

### Hadrian’s Villa archaeological site (Tivoli, Italy), the western substructures of the Canopus, and sampling

**Fig. 1.**
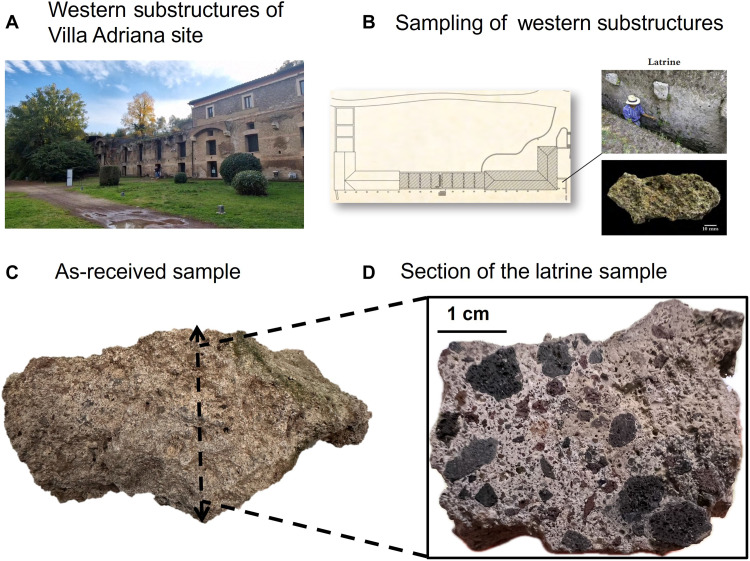
Villa Adriana (Tivoli, Italy, 2nd century AD by the Roman emperor Hadrian) is a world heritage site (UNESCO, 1999). (**A**) Photograph and (**B**) map of the sampling region (Canopus western substructure) in Hadrian’s Villa. (**C**) Actual sample. (**D**) Section of the as-received sample. (A) Photo courtesy of Istituto Villa Adriana e Villa d’Este.

Hadrian’s Villa (27 km east of Rome), a UNESCO World Heritage site since 1999 (*15*, *16*), served as Emperor Hadrian’s retreat and stands as one of the most elaborate and sophisticated architectural achievements of the Roman Empire. Among its many buildings, the western substructures of the Canopus consist of 22 residential and service rooms situated against the western slope of the so-called Valley of the Canopus, one of the most iconic places in Hadrian’s Villa (Fig. 1A). This luxurious banquet venue, articulated around a pool surrounded by statues, pergolas, and elegant architectures, obviously required service structures, which were housed in the western substructures. Within this building complex, one of the two communal latrines, located in the north area (Fig. 1, B and C), provides a unique opportunity to study the construction and material used in inland Roman concrete.

A sample was taken from the concrete collector underneath the toilet seat of the latrine, linked to the sewage system (Fig. 1C). A section of the concrete (Fig. 1D) was extracted, revealing its composite structure, with visible inclusions of volcanic aggregates and a lime-based binder matrix.

### Provenance of the major aggregate and its composition

**Fig. 2.**
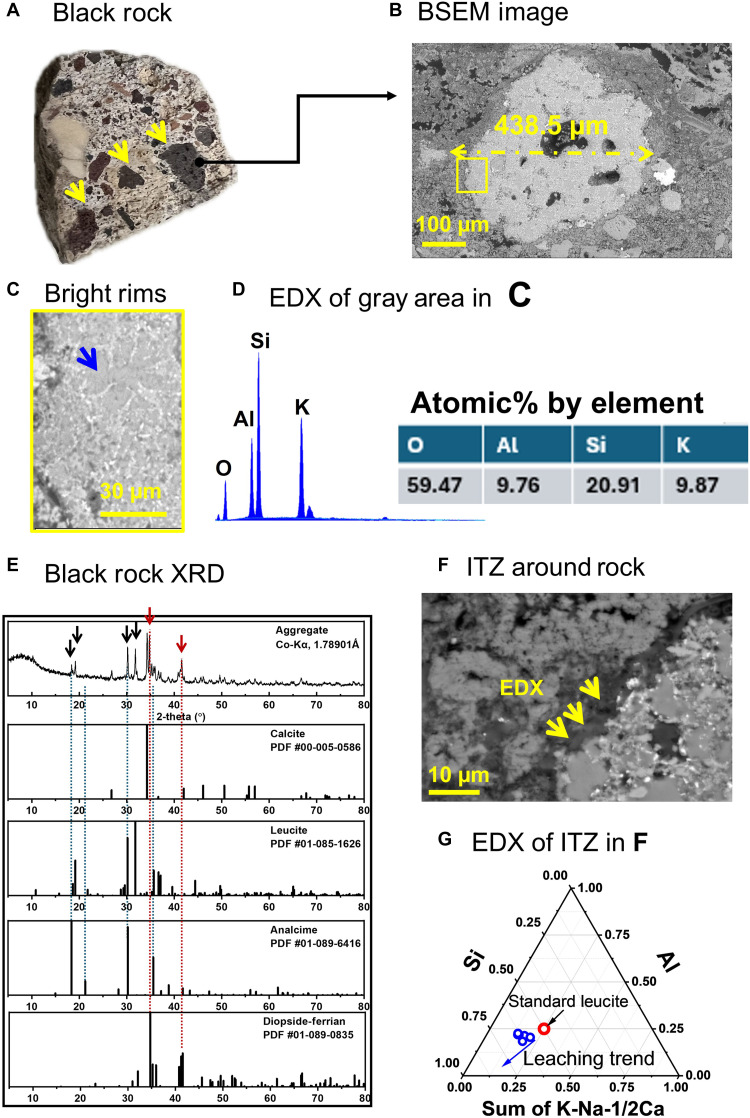
Aggregate used in the latrine sample. (**A**) Visually observed black rock and (**B**) BSEM image. (**C**) Enlarged BSEM image of aggregate with the highlight of the bright rim and (**D**) EDX of the gray area in (C). (**E**) XRD patterns of the separated black lava (Co-Kα, 1.78901 Å) with standard PDF cards. The trace differences of leucite and analcime in XRD and the ratio of the minerals in black rock can be found in fig. S2. (**F** and **G**) Morphology (F) and composition (G) of ITZ of the rock and binder.

It is well known that various volcanic rocks, including tuffs, pozzolana, and lavas, were integral to the foundations of ancient Roman architecture (*2*). Understanding the properties and roles of these volcanic materials in Roman concrete enhances archaeological knowledge and offers insights into sustainable building practices. Like other Roman concrete structures (*1*, *3*, *8*, *17*), the Latrine Canopus concrete also used black volcanic rock as its primary aggregate, highlighted by yellow arrows in Fig. 2A. Observations with BSEM reveal that the black rock contains a notable porous structure (Fig. 2B), with brighter rims uniformly distributed within the gray matrix. A closer inspection of the aggregate at a higher magnification (Fig. 2C) shows that these bright rims form vein-like lines within the rock, indicating the slow cooling process characteristic of volcanic lavas. An energy-dispersive x-ray (EDX) spectrum (Fig. 2D) of the gray matrix in Fig. 2C (blue arrow) reveals the chemical composition of the rock, showing a potassium (K):aluminum (Al):silicon (Si) atomic ratio of ~1:1:2. Further analysis using Co-Kα x-ray powder diffraction (Fig. 2E) identifies the crystal phases in the black lava, clearly indicating that the major mineral components of this aggregate are calcite [calcium carbonate (CaCO_3_)], leucite (KAlSi_2_O_6_·*n*H_2_O), analcime (NaAlSi_2_O_6_·*n*H_2_O), and ferrian diopside [Ca_1.0_(Mg_0.8_Fe_0.2_)((Si_1.7_Fe_0.2_)O_6_)]. Calcite was not observed in the aggregate’s BSEM-EDX analysis, likely because it was introduced during the sampling process when the aggregates were manually separated from the matrix. Combining the x-ray diffraction (XRD) and EDX analyses, it is evident that the gray matrix in Fig. 2C is composed of leucite, characterized by a K:Al:Si atomic ratio of 1:1:2. In addition, the EDX analysis of the brighter rim (fig. S1) reveals a significant presence of Fe and Mg, consistent with the composition of diopside.

### Interfacial transition zone (ITZ) between the binder and the aggregate

A distinct interfacial transition zone (ITZ) between the aggregate and the binder is observed in the BSEM image at a high magnification (Fig. 2F and fig. S3), a feature commonly found in concrete (*18*). The ITZ is considered the weakest region in modern concrete because of its higher porosity and defects at the interface between the hydrated cement matrix and the “inert aggregate” (*19*). However, using volcanic rocks (*6*–*8*) or ceramics (*20*) as the aggregate in Roman concrete shows clear evidence of reactions and integration with the hydrated binder matrix (*21*). Similar findings have also been confirmed by the BSEM-EDX analysis. A comparison of the ITZ composition with standard leucite (Fig. 2G) reveals notable leaching of Si and Al from the rock. The binder provides sufficient calcium ions and an alkaline environment, enabling a local reaction between aluminosilicates and calcium. This reaction generates cementitious binding phases, primarily calcium aluminosilicate hydrates (C-A-S-H), which are also the main binding phase in modern concrete (*22*). The formation of C-A-S-H around the ITZ undoubtedly enhances the mechanical stability and reduces the permeability of Roman concrete (*20*), contributing to the enhanced durability of Roman structures (*23*).

### Binder of the Latrine Canopus concrete

**Fig. 3.**
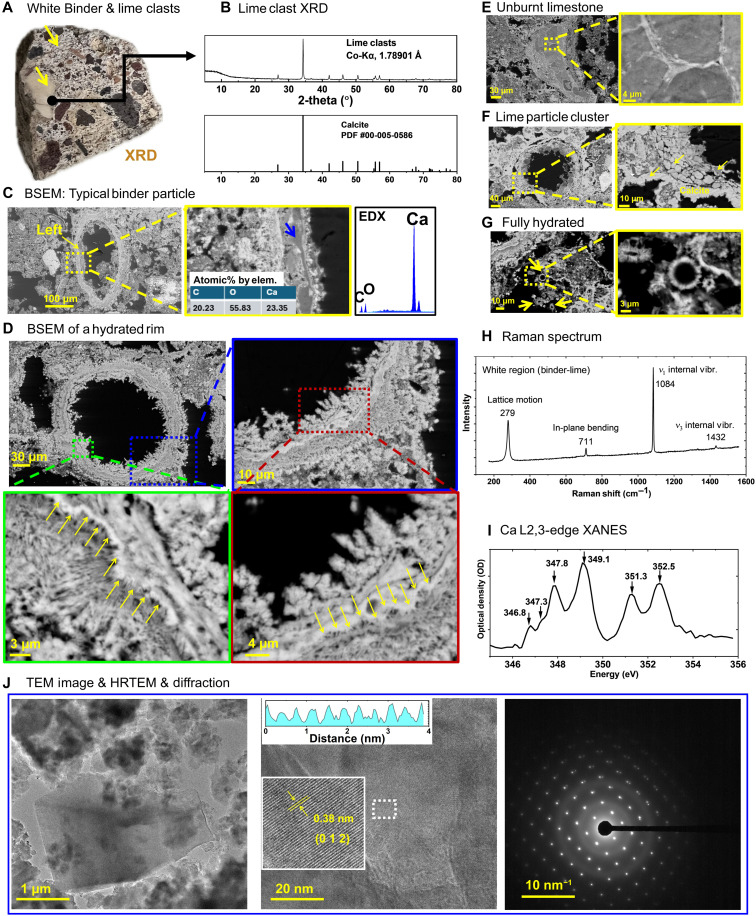
Binder used in the latrine sample. (**A**) Visually observed white binder and lime clasts and (**B**) XRD pattern. (**C**) A typical binder particle was observed under BSEM, and the chemical composition was determined (EDX). (**D**) Reaction rim of a fully hydrated particle. (**E** to **G**) Classification of the binder particles commonly observed under BSEM, including (E) unburnt limestone, (F) lime particle cluster, and (G) fully hydrated particle. (**H** to **J**) Spectroscopy analysis (other than XRD) confirming the crystal type of the main binding phase, including (H) Raman spectrum, (I) Ca L-edge XANES, and (J) TEM, HRTEM, and diffraction pattern. The crystal face assignment can be found in fig. S8.

In concrete, the binder refers to the reactive components that undergo hydration or alkali activation to form solid hydration products, which bind aggregates and provide mechanical strength. Roman builders widely used lime (burnt limestone) and pozzolans as the primary binder in their concrete, a practice identified in several iconic structures (*9*, *24*). In the case of Latrine Canopus concrete, the hydrated quicklime and coarse pozzolans were also found to be the original binder, and the quicklime part exhibits two distinct morphological characteristics, as indicated by yellow arrows in Fig. 3A. The relict lime clasts and the white hydrated binder (i.e., mortar matrix) were separated from the concrete and examined by XRD (Fig. 3B and fig. S4), which reveals that the only phase present in the relict lime clasts is calcite (Fig. 3B). In contrast, the mortar matrix primarily contains calcite and other mineral phases from the aggregates (i.e., fine pozzolans that cannot be separated) (figs. S4 and S5). The use of lime and pozzolans as the primary binder has also been confirmed in other archaeological sites, including Privernum (*9*) and the Aqua Traiana aqueduct (*25*, *26*), where carbonated hydrated lime (calcite) and C-A-S-H formed from the lime-pozzolan reaction are identified as the binding phases (*8*).

Figure 3C shows a typical hydrated binder particle, which appears nearly empty with a prominent residual rim exhibiting distinct morphologies, including fibrillar and micritized grain features. EDX analysis (Fig. 3C and fig. S6) confirms that these phases are predominantly calcium carbonates, consistent with the XRD results. Notably, some micritized grains of calcite within the rim appear to have been removed, leaving behind a “hollow-like” edge (Fig. 3C and fig. S7). In addition, a fully reacted region is shown in Fig. 3D, where the reaction rim (indicated by yellow arrows) has developed hydration products both internally and externally.

Figure 3 (E to G) classifies the lime clasts identified in the Latrine Canopus concrete, which will be discussed in detail in the following section. Further analytical spectra of random binder surfaces and powders reaffirmed that the calcium carbonate is calcite. XRD identifies calcite as the only clearly crystalline Ca-bearing phase, but XRD is less sensitive to amorphous or nanocrystalline components. Therefore, Raman spectroscopy, Ca L-edge x-ray absorption near-edge structure (XANES) spectroscopy, and transmission electron microscopy (TEM) were performed to check for possible amorphous Ca phases and to verify that the observed particles are well-crystallized calcite with characteristic spectral and diffraction signatures. Raman spectroscopy (Fig. 3H) showed the characteristic molecular motion (279 cm^−1^), bending (711 cm^−1^), and vibration (1084 and 1432 cm^−1^) of calcite (*27*, *28*). The Ca L2,3-edge XANES spectroscopy of calcite (Fig. 3I) provided insights into the oxidation state and coordination environment of calcium, confirming that the signature peaks of calcite at the Ca L3-edge (347.8 eV) and Ca L2-edge (351.3 eV) appear because of the degeneracy loss of the 2p orbitals with an energy difference (Δ*E*) of 3.5 eV (*29*, *30*). In addition, crystal diffraction and high-resolution TEM imaging (Fig. 3J) revealed distinctive features of calcite, including the crystal plane (0 1 2) and a selected area diffraction pattern that aligns well with the standard diffraction pattern (fig. S8). This evidence demonstrates that the binder in the Latrine Canopus concrete consists of lime clasts in multiple distinct states, which is a mixture prepared from burnt limestone. Over millennia, however, these lime clasts have fully transformed into calcite regardless of their original state.

### Classification of phases in quicklime prepared from burnt limestone

Although almost all the calcium oxide (CaO) phases (from quicklime) in the concrete have transformed into calcite, three major morphological categories can still be identified (Fig. 3, E to G). The first category includes unburnt or partially burnt limestone particles, which retain the characteristic micritized ooid grain morphology of the original limestone (Fig. 3E). Around these micritized grain features, brighter rims are observed, indicating a higher density of calcite (*31*). This suggests that the limestone was only partially burnt, preserving much of the micritized grain structure, while the crystalline boundaries were decomposed into lime, eventually carbonating and turning into calcite. Alternatively, these brighter rims could be the calcite cement that formed originally in the limestone rock (*31*).

The second type consists of particles that have accreted into clusters, with a distinct hydration rim formed around them (Fig. 3F). It is likely that the internal CaO particles were not fully hydrated but were carbonated once hydration ceased. As a result, these particles remain intact, with small gaps between them and their surrounding particles.

The third type consists of fully hydrated particles (Fig. 3G), typically small (<5 μm), which have fully reacted with the surrounding water. These particles usually form a hollow structure with a spherical shape (*32*). However, these fully hydrated spherical hollow structures differ from the hydration rims shown in Fig. 3D. In the smaller particles, there is almost no internal growth of carbonated hydration products (calcite). In contrast, the hollow structures in the larger hydration rim exhibit a notable amount of internal growth of reaction products.

The reaction rims of many lime clasts consist of radiaxial fibrous calcite, forming outward-growing fibrous overgrowths that locally fill adjacent pores and microcracks (*33*). These features reflect dissolution-precipitation during the gradual carbonation of residual lime phases. Although superficially similar to microstructures reported in some concretes interpreted as “hot-mixed” (*9*), our observations do not provide direct evidence for deliberate hot-mixing. Instead, the textures are more conservatively explained by incomplete burning, partial slaking, and long-term in situ carbonation, consistent with the range of lime-clast behaviors documented in inland Roman concretes.

### 3D structure of latrine concrete: Black lava and surrounding binding phases

**Fig. 4.**
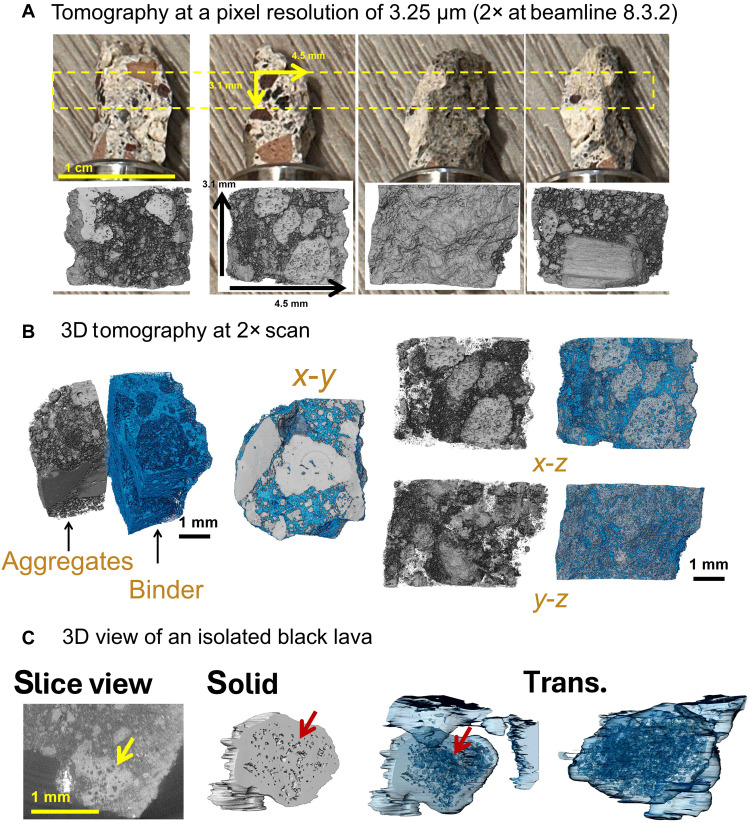
μCT scan of a latrine sample at a 2× magnification (2D pixel sizes of 3.25 μm). (**A**) 3D reconstruction of a cylindrical latrine concrete sample and its photograph. (**B**) Segmentation of aggregates and binders in the sample, together with the three-view drawing. (**C**) Isolated black lava (pozzolanic aggregates; porosity, ~15.65%) from the tomography scans from the slice view to solid parts and the internal porous structure (indicated by the yellow and red arrows) with the transparent solid phase. The 3D view videos of the black lava (aggregate) can be found in the Supplementary Materials (movies S1 to S3).

Figure 4 presents a comprehensive three-dimensional (3D) microstructural analysis of a Roman Latrine Canopus concrete sample using μCT. The photographic and reconstructed images reveal the heterogeneous composition of the concrete, with coarse black lava aggregates embedded within a porous binder matrix (Fig. 4A). The aggregates exhibit irregular shapes and sizes, highlighting the nature of volcanic lava, while the binder shows areas of dense hydration products interspersed with microporosity. The segmentation of aggregates and binders in three orthogonal planes (*x*-*y*, *x*-*z*, and *y*-*z*) provides valuable insights into the spatial distribution and interaction of these components (Fig. 4B), which are critical for understanding the relative positions of binding phases and aggregates in the concrete.

Further analysis of isolated black lava aggregates shows a porosity of ~15.6%, with interconnected voids forming a well-defined porous network (Fig. 4C). The transparent 3D visualization of the aggregate highlights its internal structure and the strong interfaces formed with the surrounding binding matrix, suggesting its active role in improving the long-term durability of the concrete. The distinct hydration rims (ITZ in fig. S3 and Fig. 2F) observed in some areas further show chemical interactions between the binder and the aggregate resulting from the pozzolanic reaction. This 3D structure illustrates the intricate microstructure of Roman concrete, where the careful integration of natural volcanic aggregates and lime binders has resulted in a robust material capable of withstanding millennia of environmental exposure. In addition, with known porosity and composition, the density of volcanic lava can be estimated on the basis of the relative volume fraction (text S2). According to Vitruvius Pollio’s *Ten Books on Architecture* (*1*), Roman engineers typically used a mix ratio of 1 part lime binder to 3 parts pozzolana aggregate for terrestrial construction, largely driven by economic considerations. However, in the case of latrine concrete, the ratio appears to be closer to 1:2, providing a reasonable estimate of the water-to-binder ratio at ~0.43 (text S2).

### 3D structure of reaction rim, original CaO cluster, and unburnt limestone cells

**Fig. 5.**
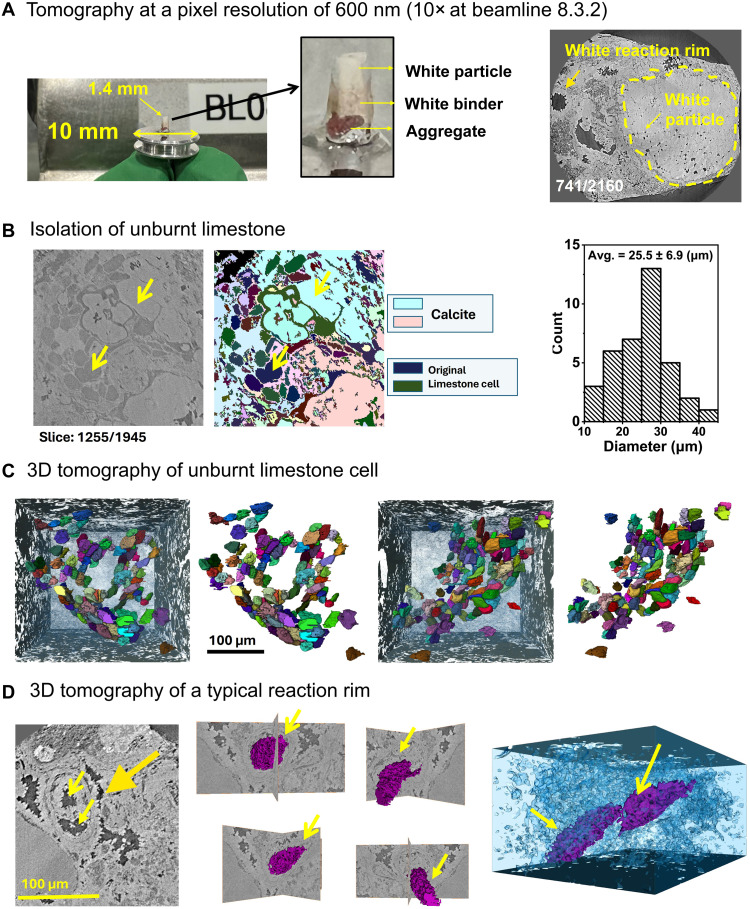
Sampling from the latrine concrete and a slice from 10× CT scan μCT (2D pixel sizes of 600 nm). (**A**) White particle, i.e., the cluster of preserved original CaO, being carbonated into calcite. (**B**) Unburnt limestone cell and its particle size distribution (*n* = 37). (**C**) Isolated 3D tomography of unburnt limestone cell. (**D**) The reaction rims from 10× CT scan and the videos of 3D structures of unburnt limestone cell in (C) and reaction rim in (D) are given in the Supplementary Materials (movies S4 to S6).

The microstructural analysis of latrine concrete using 10× magnification μCT provides a higher resolution (2D pixel size of 600 nm) to closely examine the binding phases (Fig. 5). As these lime clasts have undergone complete carbonation over two millennia, it is not possible to distinguish whether they originally existed as CaO or Ca(OH)_2_ (calcium hydroxide); therefore, we refer to them broadly as lime-derived particles that subsequently transformed into calcite. Sampling from the concrete and a μCT slice reveal a preserved white particle, identified as a cluster of lime clasts derived from burnt limestone (Fig. 5A), which has undergone carbonation with external CO_2_ and moisture to form calcite (Fig. 3B). This highlights the transformation of reactive lime phases to carbonates within the binder matrix over time, even though the CaO phase in quicklime was not fully hydrated after mixing. Unburnt limestone cells (micritized ooid grains shown in BSEM) are mapped in detail, with their spatial distribution and morphology (Fig. 5, B and C, and movie S4) shown alongside a histogram of particle size distribution (*n* = 37). The results indicate an average diameter of ~25.5 ± 6.9 μm, revealing the range of particle sizes in the unburnt limestone cells, which contains the geological information and type of the original limestone used in the latrine concrete (*31*). The ooid-type cell is a distinctive feature of oolitic limestone, which forms in shallow marine environments, as classified by Folk and summarized by Flügel and Munnecke (*34*). Roman engineers likely selected oolitic limestone for its high purity and excellent reactivity. Its consistent calcite composition and fine granularity facilitate uniform decomposition, making it ideal for producing quicklime (CaO). The oolitic limestone used for the lime clasts is consistent with high-purity limestones quarried in the region surrounding Rome (*35*) and likely provided the raw material for the quicklime found in the latrine concrete (*36*).

Reaction rims identified in the 10× CT scans reveal the intricate 3D structure of chemical reaction rims (Fig. 5D and movies S5 and S6), which closely resemble those observed in BSEM images (Fig. 3D). These rims, visualized at a higher resolution, prominently feature fibrillar calcite binding phases. Unlike the hollow structure observed in 2D images, the 3D reconstructions clarify that the hollow appearance in 2D is merely a slice of a more complex 3D particle.

Similar hollow-structure features have been observed in modern cement hydration processes (*32*). However, as noted by Richardson (*37*), these structures often contain material unless they are very small, fully hydrated particles (as seen in Fig. 3G). This interpretation is supported by the 3D structure of the reaction rims shown in Fig. 5D, where two notable holes (highlighted in solid purple) are evident. These holes demonstrate that the 2D slice showing a reaction rim is merely a cross section of much larger voids. The high-resolution μCT scans allow for detailed observation of these binding phases in three dimensions, offering valuable mechanistic insights into the chemical evolution of quicklime and their role in the formation of durable Roman concrete.

## DISCUSSION

### Binding mechanism

**Fig. 6.**
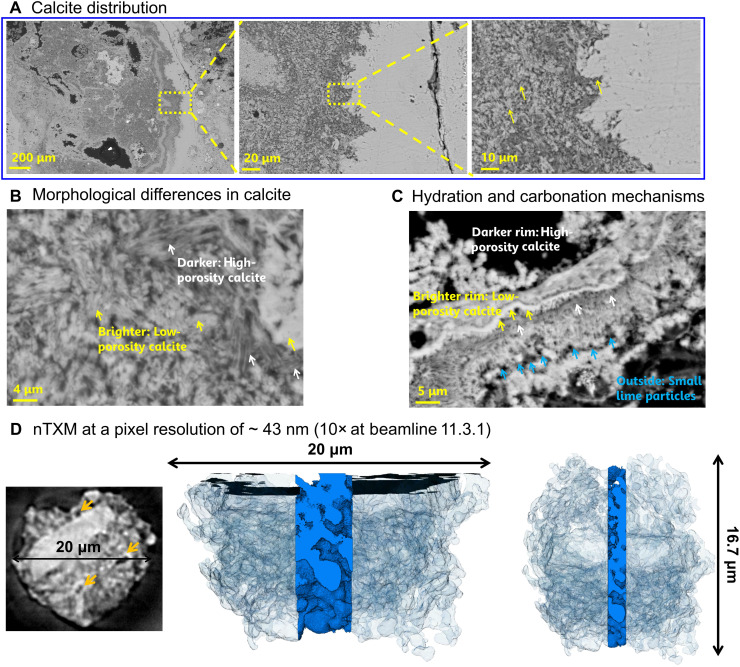
Calcite formation and CaO hydration mechanism. (**A**) Differences in calcite morphology and distribution. (**B**) High-porosity (white arrow) and low-porosity (yellow arrow) calcite. (**C**) A typical hydration rim illustrates hydration and carbonation mechanisms, i.e., radiaxial fibrous growth calcite. (**D**) Nano–transmission x-ray microscopy (nTXM) at a 2D pixel size of 43 nm (sample size, ~20 μm). The nanotomography of the 3D calcite network can be found in movies S7 and S8.

μCT and nano-CT imaging in this study is used solely to characterize the 3D morphology, connectivity, and porosity of the reaction rims and associated calcite network; these techniques do not allow mineral-phase discrimination. The identification of C-A-S-H is therefore based on BSEM-EDX analyses and bulk powder XRD, which show that C-A-S-H occurs mainly within the ITZs adjacent to volcanic aggregates. In contrast, the fibrillar networks visualized by nano-CT correspond predominantly to calcite, consistent with the Raman, XANES, and TEM observations indicating well-crystallized CaCO_3_ as the principal binding phase. The BSEM images and nano-3D structure in Fig. 6 illustrate the complex mechanisms of calcite formation and its role in binding Roman concrete. Calcite cement forms through hydration and carbonation processes, facilitated by distinct microstructural features such as reaction rims, overgrowth of calcite on preexisting calcite surfaces, and porosity variations (*33*, *38*). High-resolution μCT (Fig. 5) and nanotomography reveal the hierarchical structure of calcite, showcasing zones of high- and low-porosity calcite (yellow and white arrows in Fig. 6B), which play critical roles in concrete durability and mechanical integration.

The hydration-carbonation mechanism begins with the hydration of CaO particles, followed by carbonation in the presence of atmospheric CO_2_ (*39*). The process results in the formation of dense calcite layers, as seen in the reaction rims, which provide robust mechanical support and prevent crack propagation. The growth of calcite nucleated on existing calcite rims, where new calcite crystals seamlessly grow on preexisting substrates (rims), enhances structural coherence (Fig. 6C) (*40*, *41*). Revisiting the details of this hydration rim reveals that the small, Ca-rich particles derived from hydrated lime (indicated by blue arrows in Fig. 6C) are distributed around the larger reaction rim. Their dissolution and carbonation supply calcium ions to the surrounding solution, facilitating further calcite precipitation and overgrowth on the rim (*42*). Consequently, radiaxial growth calcite gradually fills the gaps between the hydration rim and the surrounding small particles, creating a dense and cohesive structure, which further contributes to the durability and strength of Roman concrete by forming a seamless crystal structure that enhances mechanical integrity and chemical stability. During radiaxial growth, new calcite crystals align with preexisting substrates, minimizing defects like grain boundaries and creating a coherent interface.

In addition, nanotomography highlights a 3D calcite network with a porosity of ~24.5% (Fig. 6D and movies S7 and S8), where the radiaxial fibrous calcite rims refine the local pore structure and create a denser, more coherent microstructure that enhances load transfer and limits pathways for fluid ingress, thereby contributing to the long-term stability of the latrine concrete. The C-S-H gel in modern concrete also exhibits a porous structure spanning nano- to microscales (*43*), with a porosity ranging from ~26% (high-density calcium silicate hydrate) to ~>36% (low-density calcium silicate hydrate) (*44*, *45*). These observations help explain the exceptional durability of Roman concrete, as the naturally mineralized calcite forms a comparable fibrillar structure over long timescales, contributing to its resilience and longevity. In addition, the calcite type in lime clasts and the binding phases imply that the Romans deliberately used high-purity limestone (such as oolitic limestone) as raw materials for producing lime as a reactive binder.

The overgrowth of calcite plays a critical role in enhancing the durability of Roman concrete by filling small cracks (see Fig. 6A) and voids within the matrix. During the carbonation process, calcium ions from hydrated lime or inner quicklime directly react with atmospheric CO_2_ to form porous calcite as shown by the nano-CT 2D image (figs. S9 and S10) and the BSEM images (Figs. 3 and 6), which precipitates in microcracks and pores. This process, known as carbonate mineralization, reinforces the structural integrity of the concrete, reduces the porosity of the matrix, and prevents the ingress of water and aggressive chemicals. Over time, the continual growth of calcite creates a self-healing effect, closing cracks (see an example in Fig. 6A) and mitigating further damage caused by environmental stresses or mechanical loads. Roman concretes such as the Canopus latrine provide rare full-scale examples of unreinforced, lime-based binders that have remained structurally serviceable for nearly two millennia, offering valuable insight into the long-term mineralogical evolution of carbonate-rich cementitious systems. The sustained stability of these historic materials does not imply superior durability under all conditions when compared with modern concretes; rather, it highlights the fundamentally different degradation mechanisms that operate in unreinforced lime-based binders. In contemporary infrastructure, reinforcement corrosion is the primary durability challenge, whereas the intrinsic stability of cement hydrates over long timescales is generally well established. Our observations therefore underscore how the progressive carbonation and recrystallisation processes documented here can contribute to the longevity of lime-based systems in the absence of steel while also informing broader discussions on low-clinker, CO_2_-reactive binder technologies without overstating direct parallels to modern structural applications.

## MATERIALS AND METHODS

### Scanning transmission x-ray microscopy (STXM) and transmission electron microscopy (TEM)

The binder region of the latrine concrete was manually separated and ground into fine powder (<75 μm) for scanning transmission x-ray microscopy (STXM) and TEM analysis. STXM was performed at the Advanced Light Source (ALS), Lawrence Berkeley National Lab (LBNL), using beamline 5.3.2 (*46*). The STXM data processing was performed on the basis of lab-based coding software STXM Reader software written by M. A. Marcus (*47*). The latrine binder powder was first dispersed in pure water, followed by preparing on 100-nm-thick SiN (silicon nitride) windows (100 μm^2^) for analysis. For XANES measurements at the Ca L2,3-edge, the samples were placed in a dry helium-filled chamber maintained at a pressure of 1/3 atm. Spectra for the Ca L2,3-edge (345 to 356 eV) were collected in line-scan mode with an energy resolution of 0.1 eV and a dwell time of 10 ms per point.

TEM was conducted using a 200-kV FEI monochromated F20 UT Tecnai equipped with a 2048 by 2048 charge-coupled device camera at National Center for Electron Microscopy, LBNL. The latrine binder powder was dispersed in ethanol, deposited onto a copper mesh, and allowed to dry before being mounted on the sample holder. To capture bright-field images, the electron dose was minimized (~11 e Å^−2^ s^−1^) to prevent beam-induced damage. High-resolution TEM was conducted on selected areas, achieving a resolution of up to 0.12 nm.

### Powder XRD

Aggregate, relict lime clasts and the binder matrix were separated from the latrine concrete and ground into fine powder (<75 μm). An additional sample containing all these components combined was also prepared. XRD analysis was performed using a Philips X’pert PRO Gonio XRD instrument (Department of Earth and Planetary Science, UC Berkeley), operated at 40 mA and 40 kV on a Spinner PW3064 stage. A Co-anode (Kα = 1.78901 Å) was used to mitigate the negative effects of iron in the aggregates during diffraction. Scanning was conducted with a step size of 0.017° and a step time of 33.593 s. Semiquantitative analysis, detailed in figs. S4 and S5, was carried out using HighScore software.

### Backscattered scanning electron microscopy (BSEM)-energy dispersive x-ray (EDX)

A slice of the latrine concrete (~1 × 2 cm^3^ cross-sectional area) was cut and impregnated with epoxy resin (EPO-TEK 301-2, US) under vacuum conditions. The impregnated samples were dried in an oven at 40°C for 24 hours. Afterward, the samples were cut and initially ground using a polishing machine, followed by sequential polishing in nonaqueous environment with alumina and diamond powders/pastes of decreasing particle size (9, 3, 1, and lastly, 0.25 μm). The polished samples were then carbon coated with a 10-nm layer using a LEICA EM ACE 600 High Vacuum Sputter Coater (Germany), with the coating thickness measured via quartz crystal resonance. The coated samples were examined using a Zeiss EVO MA10 Scanning Electron Microscope (Germany) equipped with an EDX detector (EDAX-Genesis) at the Department of Earth and Planetary Science, UC Berkeley. Quantitative elemental compositions were calibrated against standard minerals, with adjustments for the 10-nm carbon coating.

### Raman spectroscopy

The polished sample, without carbon coating, was used for Raman spectroscopy analysis using a LabRAM spectrometer at the Molecular Foundry, LBNL. Initial inspection was conducted with a 100× confocal microscope (aperture, 0.8; laser spot size, ~1 μm; fig. S11). Raman spectra were then collected in a backscattering configuration with a 532-nm laser excitation line at a power of ~5 mW.

### Computed tomography (CT)

All CT experiments were performed at ALS (LBNL), at beamlines 8.3.2 (μCT) and 11.3.1 (nano-CT). Reconstruction of CT data and video visualizations were performed using AVIZO software (Thermo Fisher Scientific, US).

μCT scans were performed at beamline 8.3.2 using synchrotron-based hard x-ray microtomography. Two optical objective lenses, 2× and 10× magnifications (Mitutoyo, Japan), were used, providing 2D pixel sizes of 3.25 μm and 600 nm, respectively. To fit the x-ray field of view, columnar samples were prepared from the latrine concrete: a 0.5 cm–by–0.5 cm–by–1 cm sample for the 2× scan (Fig. 4A) and a 1.4 mm–by–1.4 mm–by–4 mm sample for the 10× scan (Fig. 5A). With a constant current of 500 mA, white light was used for the 2× scan, with a 20-ms exposure time per transmission image. For the 10× scan, the incident beam energy was fixed at 20 keV using a multilayer monochromator (W/B_4_C, *d* = 2 nm) and an exposure time of 300 ms per transmission image. Each sample was scanned by rotation, producing 1969 2D projection images with a voxel resolution of 2560 by 2560 by 2324. Initial processing of data and building of tomograms was completed using a remotely accessed supercomputer Perlmutter available at the National Energy Research Scientific Computing Center (NERSC).

Nano-CT scans were conducted at beamline 11.3.1 using synchrotron-based full-field hard x-ray transmission microscopy and nanotomography. A calcite binder fragment was manually separated from the concrete and ground in a small agate mortar, and a ~20-μm particle was selected using a gas flow–controlled sampler. The particle was attached on the tip of a pin, which was then mounted on a nanotomography sample holder. The x-ray zone plate provided a magnification of ~20×, with flux levels of ~7 × 10^10^ photons/s at 6.8 keV, enabling photon transmission through the Roman concrete binder. This setup achieved a 2D pixel resolution of ~43 nm. A total of 512 projections were collected with 8000-ms exposures, yielding 2048 2D images at a resolution of 2560 by 2560 by 2324 voxels. Before 3D nano-CT reconstruction, the 2D CT images underwent preprocessing using a polynomial interpolation method (see fig. S9) to enhance the volume pixel resolution. This step ensured high-quality reconstruction and improved the accuracy of 3D visualizations.
