# Supplementary material for: Mineralized carbonates contribute to the millennial durability of Roman concrete
Source: Sci Adv. 2026 Jul 8;12(28):eaeb0754. doi: 10.1126/sciadv.aeb0754 (PMC13344319; doi:10.1126/sciadv.aeb0754)
Supplement: Supplementary file 1 — Figs. S1 to S11 Supplementary Text S1 and S2 Legends for movies S1 to S8 [file sciadv.aeb0754_sm.pdf]

Science Advances
AAAS

Supplementary Materials for

Mineralized carbonates contribute to the millennial durability of Roman concrete

Xiaohong Zhu *et al.*

Corresponding author: Xiaohong Zhu, xiaohong.zhu@bjut.edu.cn; Paulo J. M. Monteiro, monteiro@berkeley.edu

Sci. Adv. **12**, eaeb0754 (2026)
DOI: 10.1126/sciadv.aeb0754

The PDF file includes:

Figs. S1 to S11
Supplementary Text S1 and S2
Legends for movies S1 to S8

Other Supplementary Material for this manuscript includes the following:

Movies S1 to S8

Supplementary Figure

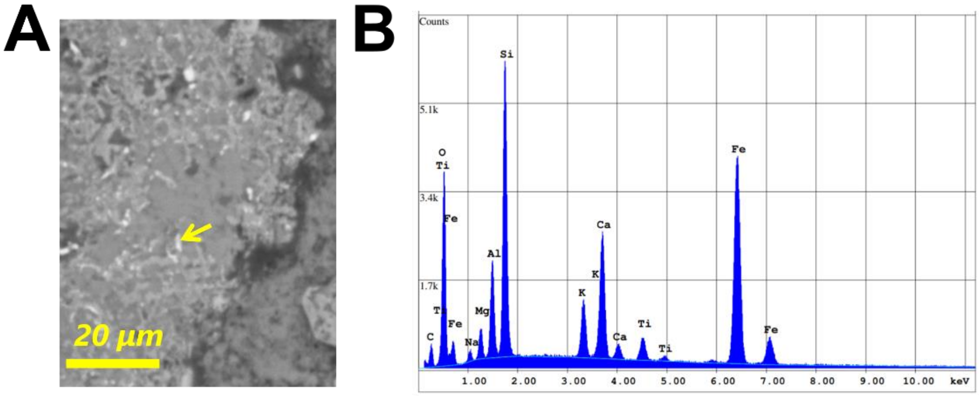

C **Atomic% by Element**

C	O	Na	Mg	Al	Si	K	Ca	Ti	Fe
8.89	56.89	1.46	2.26	4.13	10.78	1.32	3.25	0.60	10.42

Fig. S1l **Aggregate used in the Latrine sample**. Enlarged BSEM image of aggregate with the highlight of the bright rim (A) and the EDX (B) of the bright rim in (A). (C) Quantitative elemental composition (by atomic) from the EDX.

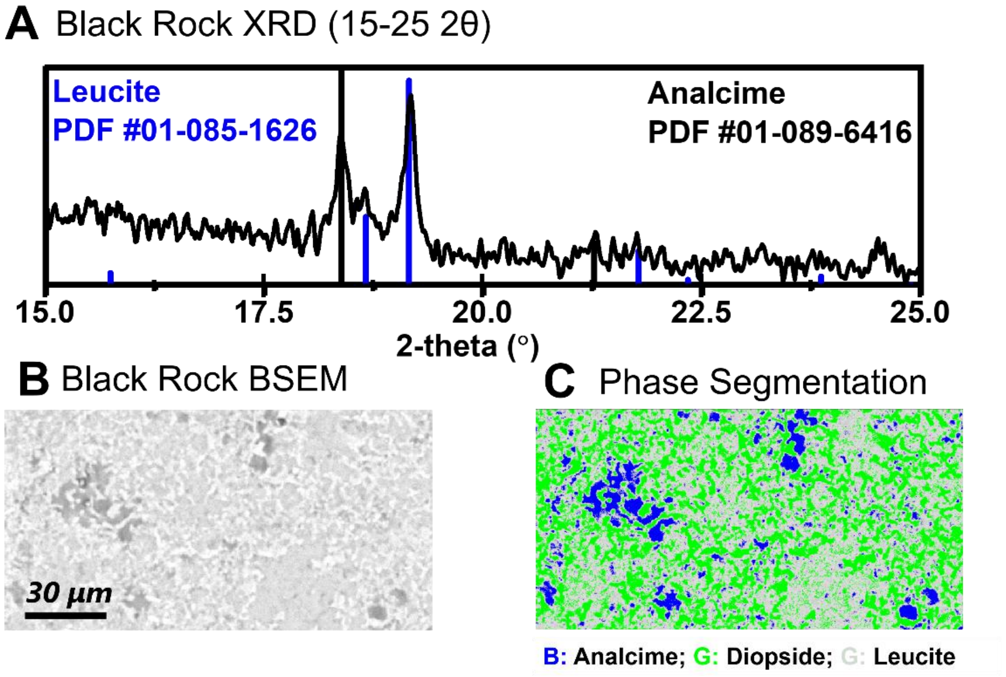

Fig. S2l **Phase compositions of aggregate**. Minor peak shifts in the XRD pattern (A), attributed to the substitution of K with Na in leucite, suggest the formation of isomorphic analcime. A representative section of the black rock's BSEM image (B) clearly shows three phases (leucite, analcime, and diopside), which can be distinguished by the compositional contrast (i.e., average atomic numbers). In this regard, the 2D volume fraction of each mineral phase can be estimated according to the segmentation of the BSEM image (C), corresponding to their mass ratio (48.6 wt.% leucite, 5.5 wt.% analcime, and 45.9 wt.% diopside-ferrian) of this lava (Text S1).

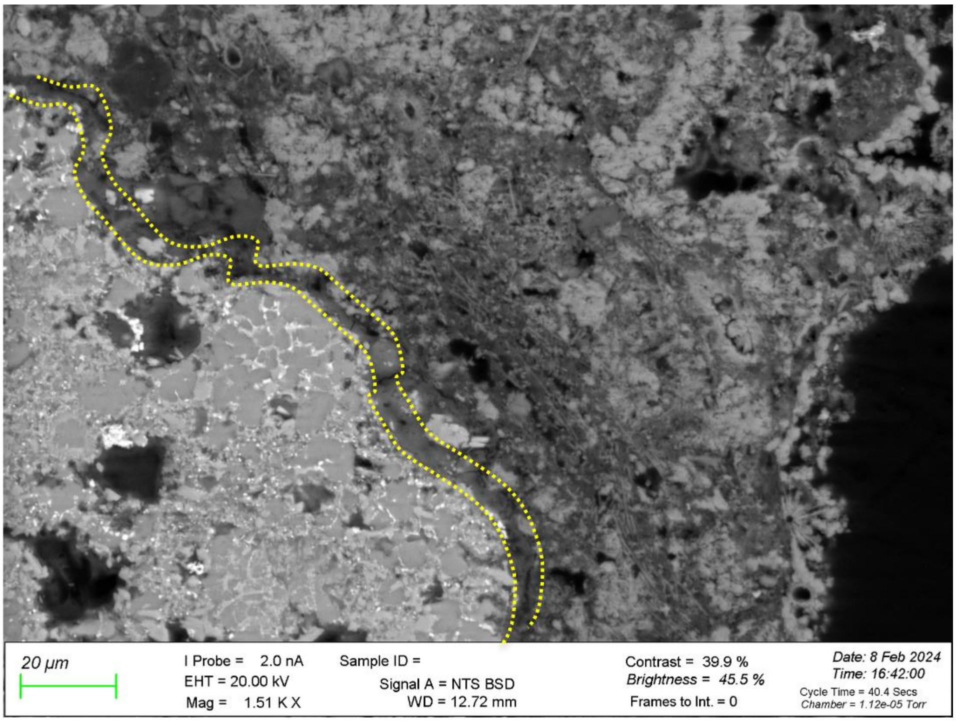

Fig. S3l **BSEM image of the ITZ between binder and black tuff aggregate.**

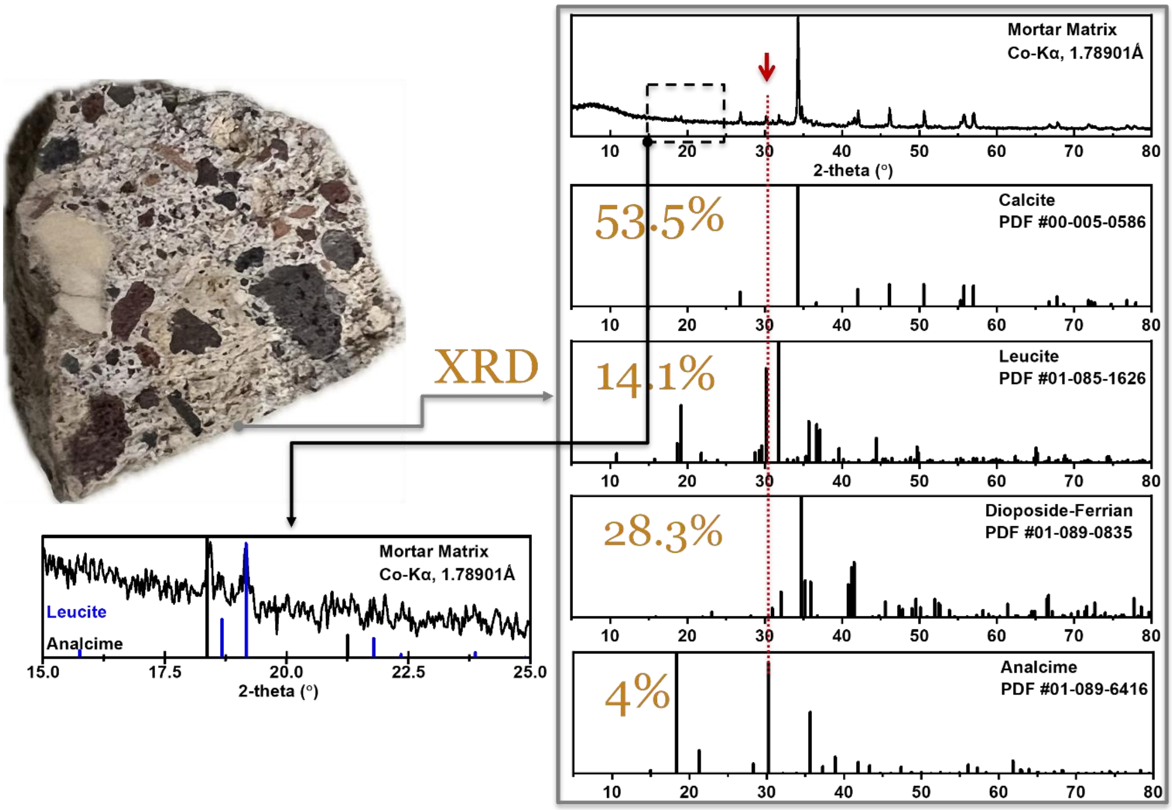

Fig. S4l **XRD pattern of the mortar matrix (white region), which contains small black lavas that cannot be easily separated.**

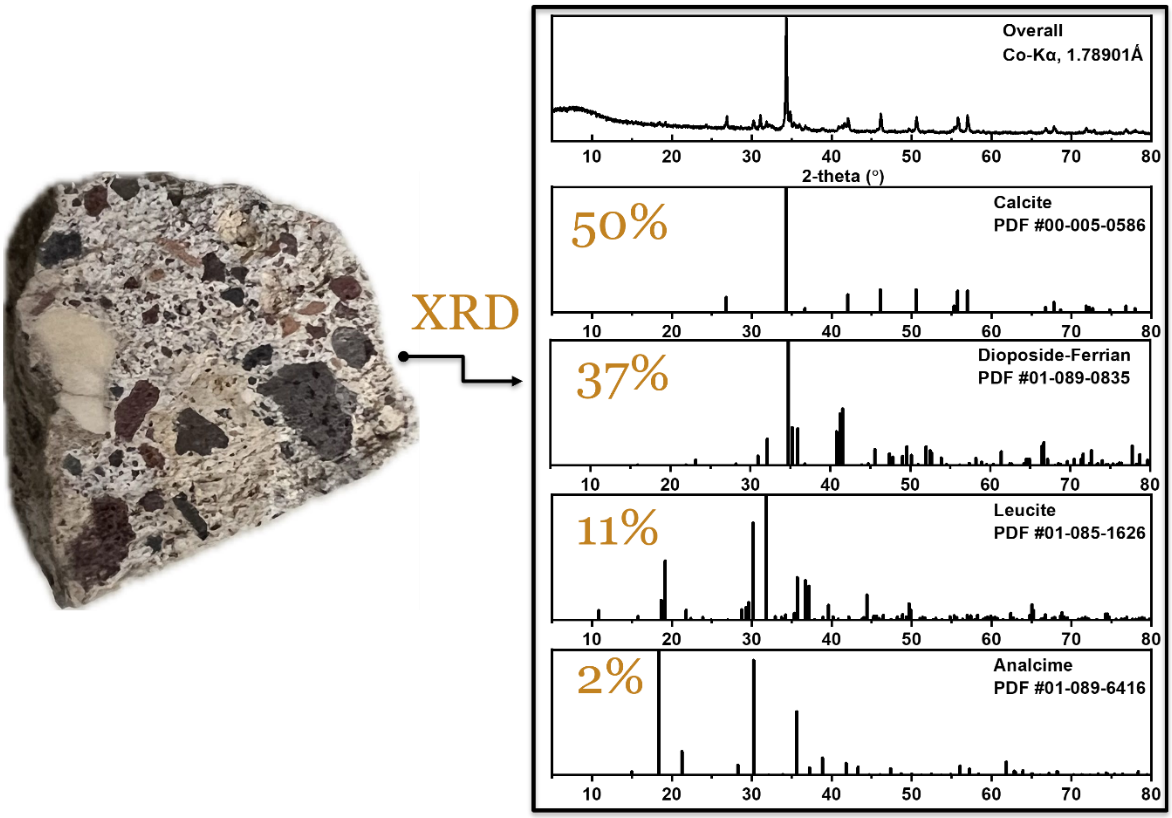

Fig. S5| **XRD pattern of the overall mortar matrix (without any separation).**

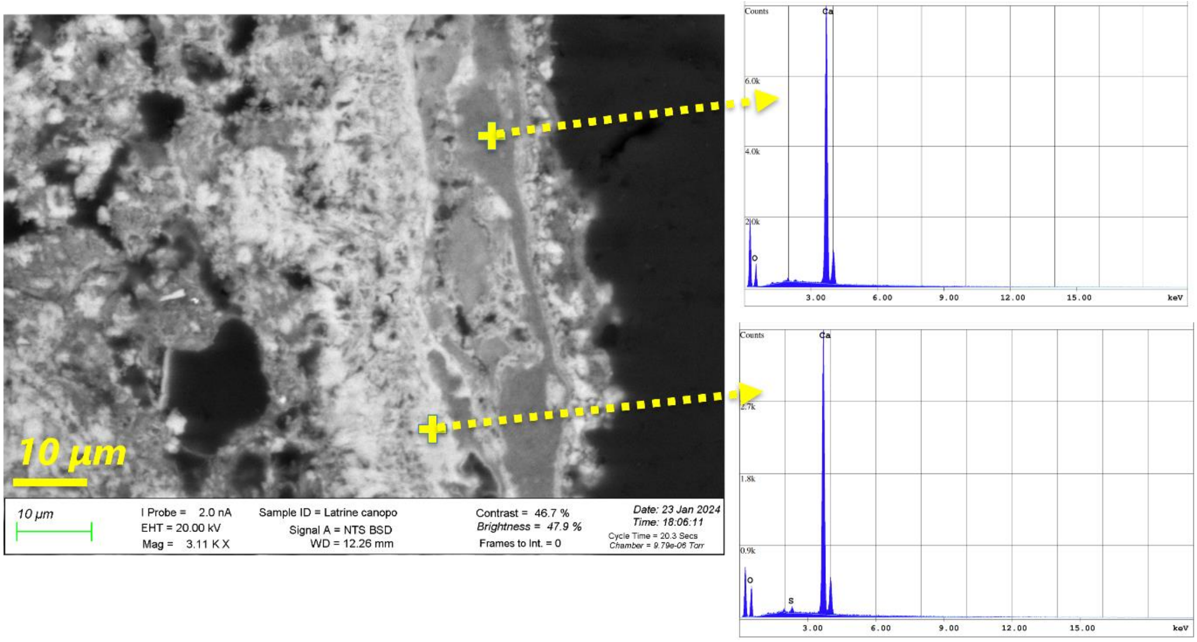

Fig. S6| **Additional EDX of calcite in Figure 3C**.

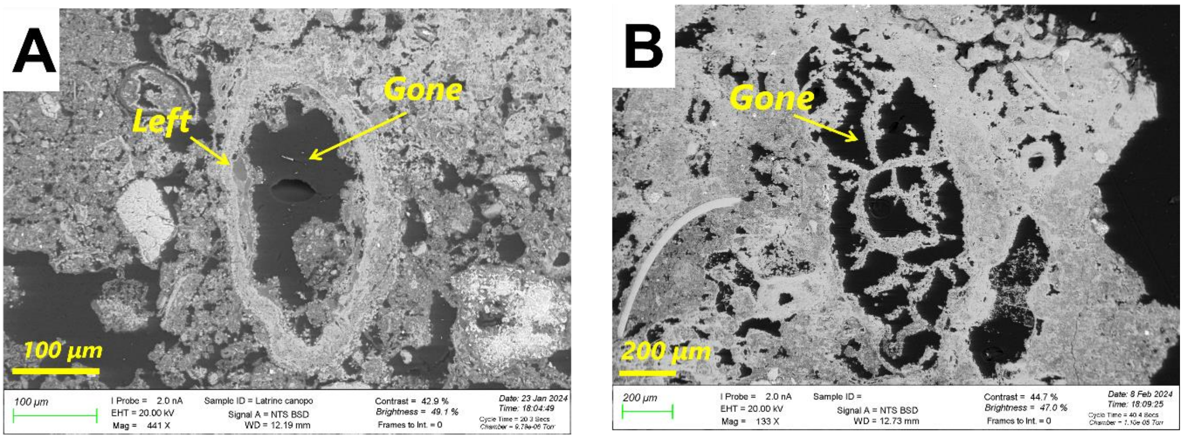

Fig. S7l **BSEM images of the hydrated lime clasts**. Some slab-like calcite left in (A) and all slab-like calcites were all removed (B).

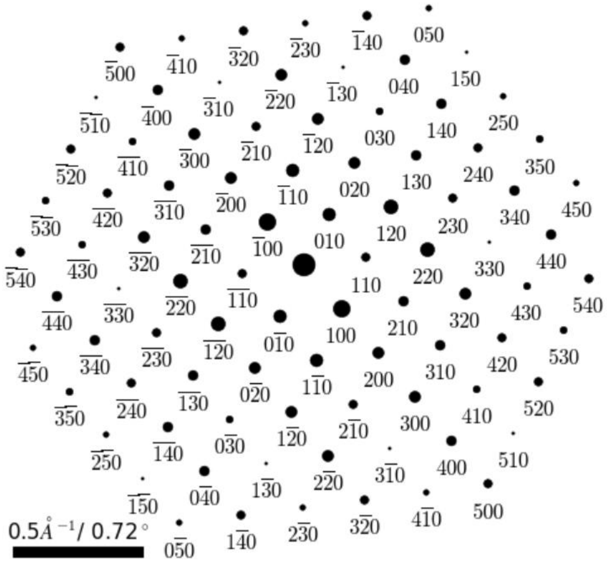

Fig. S8l **Crystal faces of diffraction pattern (TEM image in Figure 3J) of calcite**.

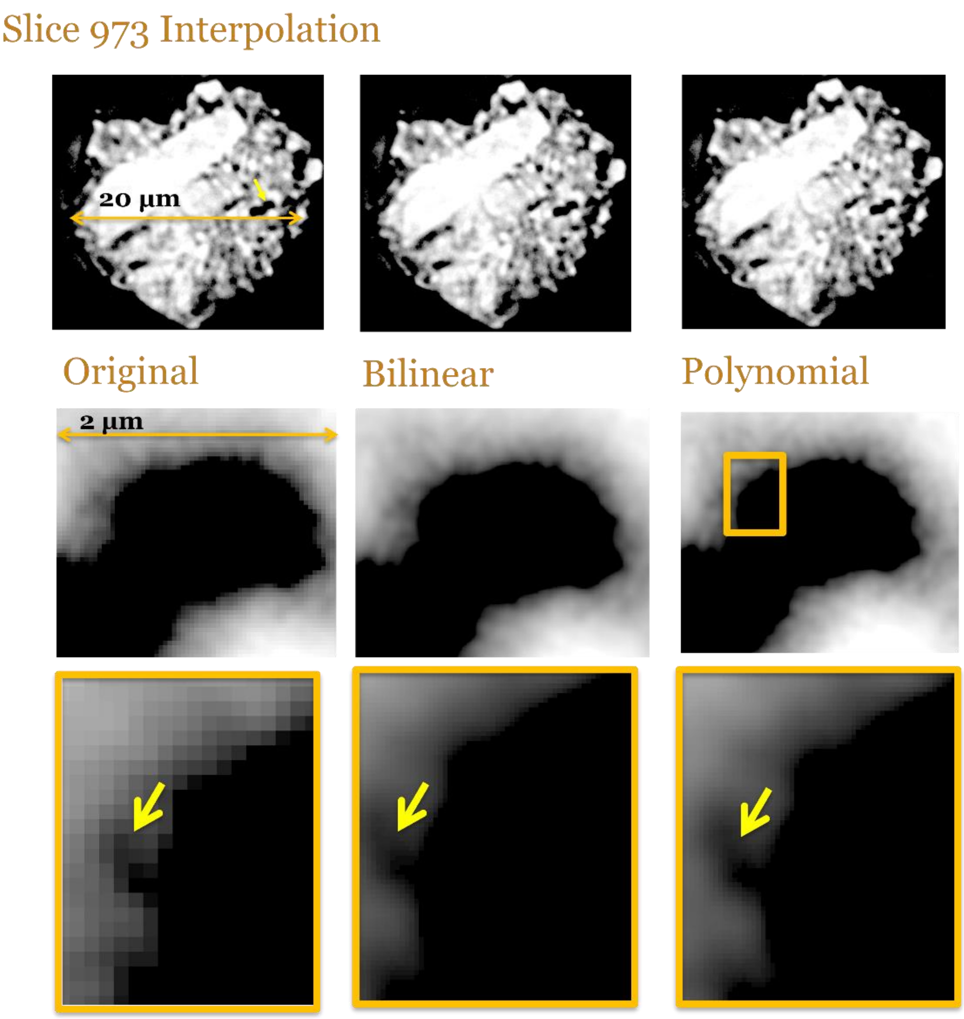

Fig. S9I **Resolution enhancement based on interpolation methods before reconstruction.** Using CT-slice 973/2048 as an example. Both methods were used and polynomial interpolation can provide a better resolution. The following 3D reconstruction (**Figure 6D** and **Supplementary Video 4**) was based on the pre-treatment of the images.

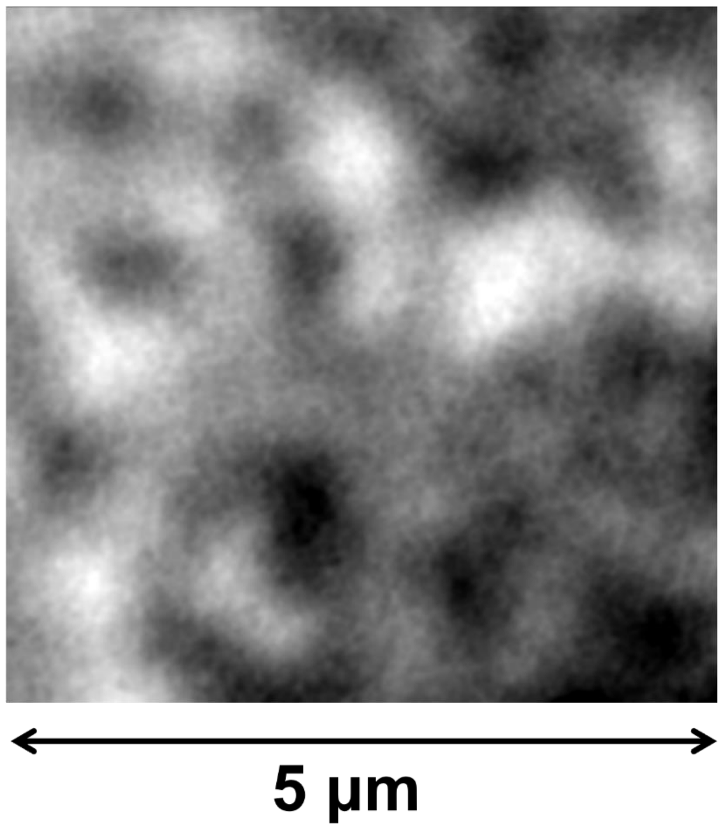

Fig. S10l **Representative area of porous calcite from 2D Nano-CT image, and the 2D volume porosity of this region is estimated as 31.5%.**

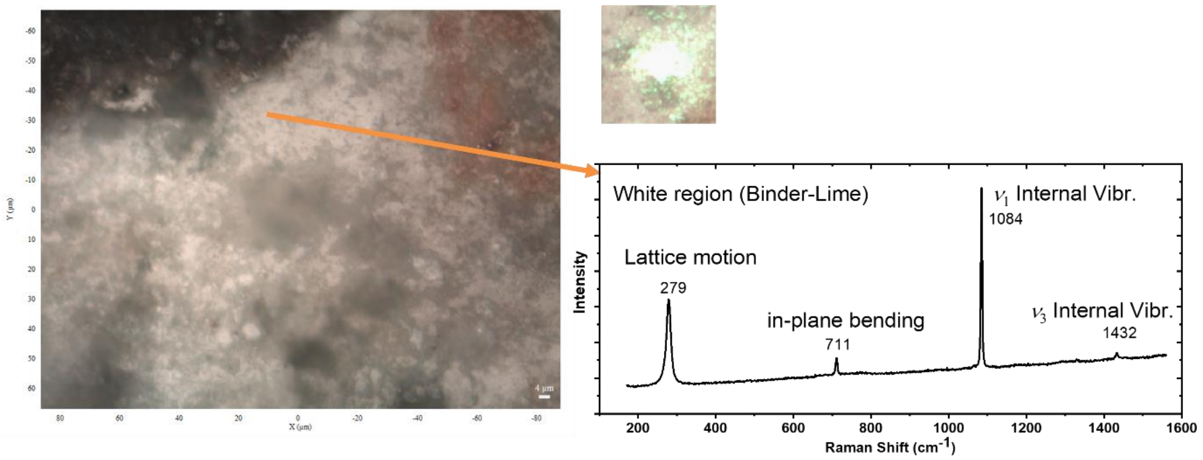

Fig. S11l **Position of Raman spectroscopy**. Optical image obtained by a confocal microscope and the focused laser beam (size: ~ 1 µm^2) on the white binder region. The Raman spectroscopy was used to check multiple white areas.

Supplementary Text

Text 1. Calculation of mineral composition based on BSEM images

Area fraction based on BSEM image (2D volume):

-- Analcime: 6.86%; Diopside-ferrian: 37.46%; Leucite: 55.68%

Density (g/cm^3) of mineral phases (data from American Mineralogist crystal structure database):

-- Analcime: 2.26; Diopside-ferrian: 3.46; Leucite: 2.47

Mass ratio of mineral phases:

-- $m = \rho v$

where *m*, ρ, and *v* are the mass, density, and volume of mineral phases.

Analcime : Leucite : Diopside-ferrian = (2.26 × 6.86%) : (2.47 × 55.68%) : (3.46 × 37.46%) = 0.155036 : 1.375296 : 1.296116

Analcime wt.% = 0.155036/(0.155036 + 1.375296 + 1.296116) = 0.155036/2.826448 = 5.48%

Leucite wt.% = 1.375296/2.826448 = 48.66%

Diopside-ferrian wt.% = 1.296116/2.826448 = 45.86%

Text 2. Estimation of water to binder ratio based on aggregate porosity

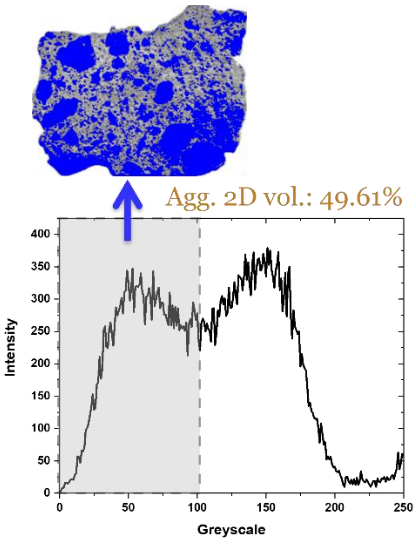

Fig. Text S1 I **Volume fraction of aggregate in Latrine Canopus concrete**.

Known: 2 parts aggregate - 1 part lime - *x*% water

Density: lime 2.21 g/cm^3

Density of agg.: 15.65% porosity

Mixed mineral density: $\rho_{avg.} = \rho_i v_i$

Mineral density: ρ = 6.86% × 2.26 + 37.46% ×3.46 + 55.68% ×2.47 = 2.83 g/cm^3 ; include the voids: 2.39 g/cm^3

Vol. of (lime + water) = 50.39%

Assume: 2 kg pozzolana; 1 kg lime and 0.03*x* kg water used

(2/2.39)/(1/2.21 + 0.03*x*/1 + 2/2.39) = 49.61%

vol.(pozzolana)/vol.(lime + water + pozzolana) = relative volume of pozzolana

x = 14.39 (total water should be 14.39% × 3 kg = 0.4317 kg)

Therefore, water/lime ratio is roughly 0.432[1].

[1] This value is an estimate derived from the mass–volume balance method, which is intrinsically associated with uncertainty.

Captions

Fig. S1l **Aggregate used in the Latrine sample**. Enlarged BSEM image of aggregate with the highlight of the bright rim (A) and the EDX (B) of the bright rim in (A). (C) Quantitative elemental composition (by atomic) from the EDX.

Fig. S2l **Phase compositions of aggregate**. Minor peak shifts in the XRD pattern (A), attributed to the substitution of K with Na in leucite, suggest the formation of isomorphic analcime. A representative section of the black rock's BSEM image (B) clearly shows three phases (leucite, analcime, and diopside), which can be distinguished by the compositional contrast (i.e., average atomic numbers). In this regard, the 2D volume fraction of each mineral phase can be estimated according to the segmentation of the BSEM image (C), corresponding to their mass ratio (48.6 wt.% leucite, 5.5 wt.% analcime, and 45.9 wt.% diopside-ferrian) of this lava (Text S1).

Fig. S3l **BSEM image of the ITZ between binder and black tuff aggregate.**

Fig. S4l **XRD pattern of the mortar matrix (white region), which contains small black lavas that cannot be easily separated.**

Fig. S5l **XRD pattern of the overall mortar matrix (without any separation).**

Fig. S6l **Additional EDX of calcite in Figure 3C**.

Fig. S7l **BSEM images of the hydrated lime clasts**. Some slab-like calcite left in (A) and all slab-like calcites were all removed (B).

Fig. S8l **Crystal faces of diffraction pattern (TEM image in Figure 3J) of calcite**.

Fig. S9l **Resolution enhancement based on interpolation methods before reconstruction.** Using CT-slice 973/2048 as an example. Both methods were used and polynomial interpolation can provide a better resolution. The following 3D reconstruction (**Figure 6D** and **Supplementary Video 4**) was based on the pre-treatment of the images.

Fig. S10l **Representative area of porous calcite from 2D Nano-CT image, and the 2D volume porosity of this region is estimated as 31.5%.**

Fig. S11l **Position of Raman spectroscopy**. Optical image obtained by a confocal microscope and the focused laser beam (size: ~ 1 μm^2) on the white binder region. The Raman spectroscopy was used to check multiple white areas.

Text 1**. Calculation of mineral composition based on BSEM images**

Text 2**. Estimation of water to binder ratio based on aggregate porosity**

Supplementary Video 1 (Movies Number 1-3): 3D view videos of the black lava (aggregate)
Supplementary Video 2 (Movies Number 4): 3D structures of unburnt limestone cell.
Supplementary Video 3 (Movies Number 5-6): 3D structures of reaction rim.
Supplementary Video 4 (Movies Number 7-8): The nano-tomography of the 3D calcite network
